# Investigating the Response of Human Neutrophils to Hydrophilic and Hydrophobic Micro-Rough Titanium Surfaces

**DOI:** 10.3390/ma13153421

**Published:** 2020-08-03

**Authors:** Karim El Kholy, Daniel Buser, Julia-Gabriella Wittneben, Dieter D. Bosshardt, Thomas E. Van Dyke, Michael J. Kowolik

**Affiliations:** 1 Department of Oral Medicine, Infection and Immunity, Harvard University School of Dental Medicine, Boston, MA 02115, USA; tvandyke@forsyth.org; 2 Center of Clinical and Translational Research, The Forsyth Institute, Cambridge, MA 02142, USA; 3 Department of Oral Surgery and Stomatology, University of Bern School of Dental Medicine, 3010 Bern, Switzerland; daniel.buser@zmk.unibe.ch (D.B.); dieter.bosshardt@zmk.unibe.ch (D.D.B.); 4 Department of Reconstructive Dentistry and Gerodontology, University of Bern School of Dental Medicine, 3010 Bern, Switzerland; julia.wittneben@zmk.unibe.ch; 5 Department of Periodontics, Indiana University School of Dentistry, Indianapolis, IN 46202, USA; mkowolik@iupui.edu

**Keywords:** hydrophilic, dental implant, biomaterials, titanium, surface chemistry, implant surface, osseointegration

## Abstract

Various treatments have been used to change both the topography and chemistry of titanium surfaces, aiming to enhance tissue response and reduce healing times of endosseous implants. Most studies to date focused on bone healing around dental implants occurring later during the healing cascade. However, the impact of the initial inflammatory response in the surgical wound site on the success and healing time of dental implants is crucial for implant integration and success, yet it is still poorly understood. The purpose of this study was to investigate the effect of titanium surface hydrophilicity on the response of human neutrophils by monitoring oxygen radical production, which was measured as chemiluminescence activity. Materials and Methods: Neutrophils were isolated from human donors’ blood buffy coats using the double sucrose gradient method. Neutrophils were exposed to both hydrophilic and hydrophobic titanium surfaces with identical topographies in the presence and absence of human serum. This resulted in six experimental groups including two different implant surfaces, with and without exposure to human serum, and two control groups including an active control with cells alone and a passive control with no cells. Two samples from each group were fixed and analyzed by SEM. Comparisons between surface treatments for differences in chemiluminescence values were performed using analysis of variance ANOVA. Results and Conclusion: In the absence of exposure to serum, there was no significant difference noted between the reaction of neutrophils to hydrophilic and hydrophobic surfaces. However, there was a significant reduction in the mean and active chemiluminescence activity of neutrophils to serum-coated hydrophilic titanium surfaces than to serum-coated hydrophobic titanium surfaces. This suggests that surface hydrophilicity promotes enhanced adsorption of serum proteins, which leads to decreased provocation of initial immune cells and reduction of local oxygen radical production during wound healing. This can help explain the faster osseointegration demonstrated by hydrophilic titanium implants.

## 1. Introduction

Dental rehabilitation of edentulous or partially edentulous patients with fixed prostheses supported by titanium implants is well accepted and documented in extended clinical trials [1,2]. The intimate contact formed between titanium surfaces and bone (osseointegration) has been paramount to the success of dental implants [3,4]. Consequently, the titanium surface used in dental implants has been modified over the past few decades in an attempt to accelerate and improve healing outcomes. Wennerberg and Albrektsson published an extensive review on the subject of surface topographies of commercially available titanium dental implants and their effect on tissue integration [5]. Surface roughness (Sa) is classified according to the degree of roughness into four categories: smooth (Sa = 0.0–0.4 μm), minimally rough (Sa = 0.5–1.0 μm), moderately rough (Sa = 1.0–2.0 μm), and rough (Sa > 2.0 μm) [6]. Moderately rough surfaces have been shown in a number of in vitro and in vivo reports to enhance the growth of osteoblasts and increase torque removal forces when compared to smoother machined surfaces [5,7,8,9,10]. Moderately rough surfaces are often hydrophobic and produced by sandblasting and/or acid etching of the titanium surface. In 2006, a manufacturer introduced a chemically-modified moderately rough (CMR) and hydrophilic version of their long-established moderately rough (MR) and hydrophobic surface. Besides being the first hydrophilic titanium surface for the application of dental implants, the new surface provides less carbon contamination and superior conditions for direct protein adsorption. In vivo studies demonstrated better clot stabilization at the early phases of healing, thus resulting in shorter healing and osseointegration times [11,12,13,14,15,16]. In vitro studies demonstrated that hydrophilic surfaces promote earlier activation of the regenerative M2 macrophage response [17,18,19,20]. Furthermore, osteoblasts demonstrated increased osteocalcin and local growth factor production when exposed to hydrophilic titanium surfaces when compared to hydrophobic surfaces [21]. Consequently, clinical recommendations for shorter loading protocols were adjusted to be at three to four weeks instead of the standard six to eight weeks. This resulted in a significantly reduced implant therapy time [22,23].

One of the possible factors contributing to reduced healing times with hydrophilic surfaces is the reaction of the initial inflammatory cells populating the implant surface immediately after being placed in the surgically prepared osteotomy site. Ivanovski et al. demonstrated that inflammation is a key biological mechanism, tightly regulated during early wound healing, which impacts osseointegration and guided bone regeneration [24]. The impact of the initial inflammatory response on the healing time and the osseointegration of endosteal implants is potentially crucial to rapid healing but is still poorly understood. The primary cellular response in inflammation comes from the polymorphonuclear leukocytes (PMNs), which include neutrophils, basophils, and eosinophils. Neutrophils are the first line of defense and are immediately present in any bleeding wound. These cells constitute 40–65% of the white blood cell population and are responsible for releasing cytotoxic products that are essential for killing bacterial invaders, but these same molecules can also destroy host tissues. Within seconds after stimulation, phagocytes show a sharp increase in oxygen uptake and begin to release large quantities of reactive oxygen metabolites. These include singlet oxygen, superoxide, hydrogen peroxide, and hydroxyl radicals.

When these toxic oxygen metabolites are released, one consequence of this respiratory burst is that light is emitted [25,26]. In 1974, Allen et al. discovered that activated neutrophils could generate light known as chemiluminescence (CL), and this forms the basis for an extremely sensitive assay that can measure reactive oxidant production by a small number of neutrophils [27]. Neutrophil CL can be detected in as little as 5 μL of unfractionated human blood [28]. Consequently, a specific range of neutrophil priming and activation to an implanted biomaterial is essential for a proper wound healing cascade and ultimate implant tissue integration. However, an exaggerated respiratory burst-centered reaction with the associated released oxygen radicals might lead to local tissue damage and delayed wound healing.

The purpose of this in vitro study was to investigate the response of human neutrophils to a hydrophobic sandblasted acid etched surface (MR) and to a chemically-modified, sandblasted, and acid-etched surface (CMR) by monitoring their chemiluminescence (CL) activity.

## 2. Materials and Methods 

Blood buffy coats were received from Indiana Blood Center (IBC) in Indianapolis, IN, USA. Institutional Review Board approval from Indiana University School of Medicine was granted (approval number NS0806-02). Buffy coats were then diluted 1:1 with Roswell Park Memorial Institute (RPMI) cell media to maximize the efficiency of separation. Neutrophils were isolated from blood buffy coats using the double dextran gradient method described by Kowolik et al. [29,30]. Cells were then counted under a hemocytometer using the trypan blue exclusion test. Cell viability was 99% for all samples.

MR and CMR treated discs were supplied by the same implant company (Straumann AG, Basel, Switzerland). Four groups were comprised of two different implant surfaces with or without exposure of discs to human serum. Two control groups included an active control with cells alone (no discs) and a passive control with reagents alone.

This resulted in a total of 6 groups as follows:

(1) Reagents alone (*n* = 24)
(2) Cells alone (*n* = 24)
(3) Cells + MR discs (*n* = 24)
(4) Cells + CMR discs (*n* = 24)
(5) Cells + MR discs after exposure to human serum for 30 min (*n* = 24)
(6) Cells + CMR discs after exposure to human serum for 30 min (*n* = 24)

### Chemiluminescence Reaction Measurement

CL assays were performed according to established protocols for human neutrophils with a chemiluminometer [31]. Titanium discs were placed in the base of a polystyrene reaction cuvette in 300 μL sterile phosphate buffer saline (PBS) and 500 μL of a neutrophil suspension containing 500,000 cells was added. In 2 treatment groups, the discs were immersed in human serum obtained during the isolation method (homologous to the cells) for 30 min before placing them in cuvettes. We added 100 μL of 1 × 10^−6^ luminol at time zero and, after 30 min, 1 × 10^−6^ M of the stimulant phorbol myristate acetate (PMA) was added. The total reaction volume was 1.0 mL. The reaction was followed for a further 60 min. The CL assays were performed in a 1251 Bio-Orbit Luminometer (Bio-Orbit, Turku, Finland). Cellular activation was recorded in millivoltages, the integrals calculated, and data analysis was performed on the mean values of triplicate experiments.

Chemiluminescence data from each experimental were divided by the control to standardize the data from different experimental runs. The CL values were summarized (mean, standard deviation, standard error, minimum, and maximum) for each group. Statistical significance was assessed using analysis of variance (ANOVA). CL data were summarized for each surface type and serum presence combination. The effects of surface type and serum presence were assessed. The ANOVA included terms for surface type, serum presence, type-by-serum presence interaction, and a random effect for experimental run. A *p*-value of <0.05 was considered significant. For post-hoc testing, the Tukey method was used for comparing all possible group pairings. Two samples of each group were immediately fixed in 5% glutaraldehyde for scanning electron microscopic (SEM, Thermo Fisher Scientific, Waltham, MA, USA) analysis. Samples were dehydrated through a graded series of ethanol, chemically dried in Hexamethyldisilazane (HMDS), attached to aluminum with epoxy for examination, sputter coated with 60/40 gold/palladium alloy, and examined at 25 kV by scanning electron microscopy.

## 3. Results

### 3.1. Chemiluminescence Activity 

Chemiluminescence measurements were used to quantify the oxidative burst by neutrophils (Table 1). Each experimental cycle resulted in three chemiluminescence measurements:

Active CL (active CL): representing the area under the CL curve, which is the integrated energy output over the 90 min total reaction period.
Mean CL (mean CL): representing the mean value of CL during the experimental run.
Peak CL (peak CL): representing the highest reading during the experimental run.

**Figure 1:**
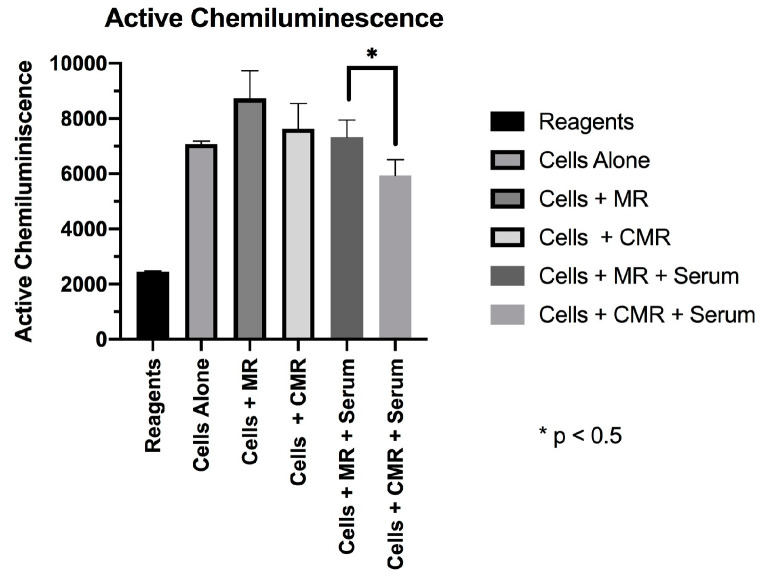
Active chemiluminescence.

**Figure 2:**
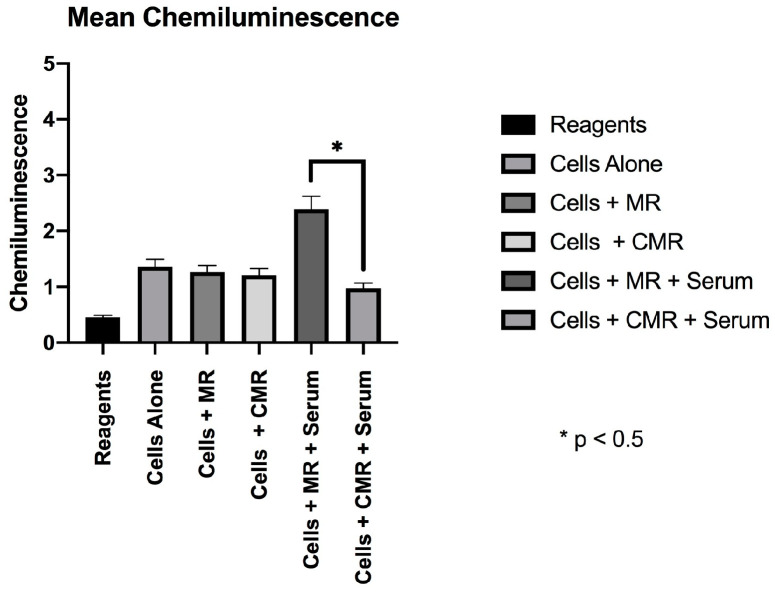
Mean chemiluminescence.

**Figure 3:**
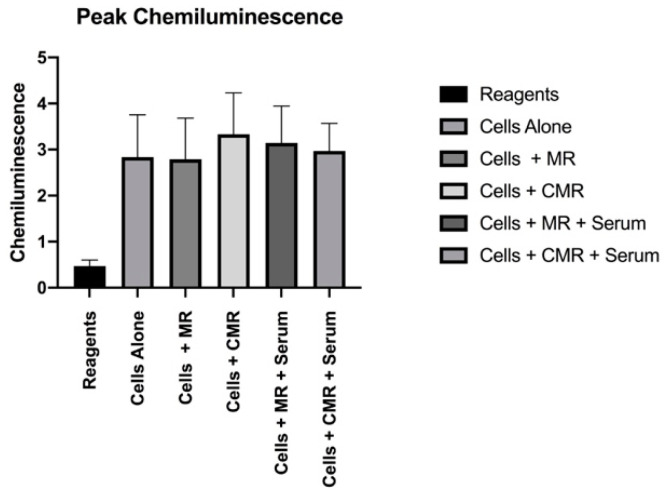
Peak chemiluminescence.

In the absence of serum coatings, MR and CMR discs did not have significantly different active CL (p = 0.35), mean CL (p = 0.29), or peak CL (p = 0.42). Serum-coated CMR discs provoked significantly less active CL (5926.227) and Mean CL (0.987) in the CMR group when compared to the Active CL (7322.863) and Mean CL (2.392) values in the serum-coated discs in the MR group. These differences were statistically significant (p < 0.05) (Figure 1 and Figure 2). No significant differences were found in peak chemiluminescence in any of the experimental and positive control groups (p > 0.05) (Figure 3).

### 3.2. SEM Imaging

**Figure 4:**
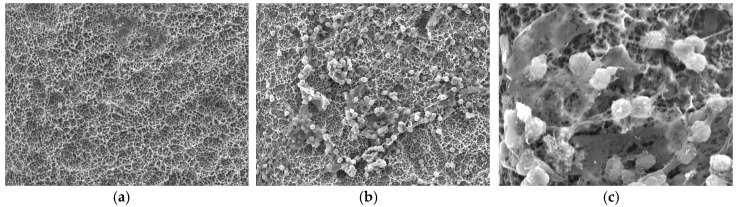
(**a**) SEM photograph at 500× magnification, showing clustered neutrophils on an MR disc, post PMA activation, with lack of cellular morphology and adaptation. (**b**) SEM image at 500× magnification showing clustered neutrophils on a CMR surface disc, post PMA activation, showing intact cellular morphology. (**c**) SEM image at 2500× magnification on a CMR disc showing intact cellular morphology with pseudopodia extending onto surface indicating normal function.

Neutrophil activation: SEM photomicrographs following 60-min incubation and immediately following stimulation with PMA (Figure 4).

## 4. Discussion

Davies et al. opined that by the time osseointegration occurs, most of the critical events in healing around dental implants have taken place [32]. The trend toward immediate and early loading of dental implants makes it prudent to understand the early healing events around the endosteal implants immediately after insertion. This understanding will help guide future research of implant surfaces and biomaterials that demonstrate faster healing and integration times. The primary stage in wound healing after the surgical placement of biomaterials is an inflammatory response. This injury and the subsequent perturbation of the homeostatic mechanisms trigger cellular cascades of wound healing. Therefore, initial inflammatory responses to endosseous titanium implants are thought to play a crucial role in forming and maintaining osseointegration, and thereby clinical success. This response is dependent on multiple factors including the extent of the injury, blood–material interactions, provisional matrix formation, the extent or degree of cellular necrosis, and the extent of the inflammatory response. The response of human neutrophils, the initial inflammatory cells populating the implant surface, may play a fundamental role in the early phases of wound healing. The level of neutrophil priming and activation may be linked to the rate and type of healing following implant placement, as well as the long-term stability of the rigid bone–implant interface. In this context, we attempted to test the response of human neutrophils to a hydrophilic microrough surface against a topographically identical hydrophobic microrough surface coated with homologous serum. The research team hypothesized that a difference in the initial inflammatory response could be at least in part responsible for this superior healing quality, previously demonstrated in in vivo trials [13,14] by influencing the trajectory of the wound healing cascade. The results suggested that the explanation behind clinically-observed healing superiority of the CMR surface implants might begin at the response of initial inflammatory cells coming into contact with the implant surface. In this study, neutrophils exposed to the serum-coated hydrophilic CMR surfaces produced less harmful oxygen radicals compared with neutrophils exposed to hydrophobic MR surfaces. A limitation of the current investigation is that no attempt was made to identify or quantify the types and nature of serum proteins attachment to the surface. Previous investigations demonstrated that the neutrophil recognition and response to biomaterials can be significantly influenced by the nature of the protein layer that coats the biomaterial surface immediately after implantation [33,34,35]. The biomaterial surface characteristics may directly influence the attraction and binding of serum proteins as well as affect the presentation manner of the adsorbed protein to inflammatory cells. The predominating proteins that bind to implanted biomaterials are IGg, albumin, and fibrinogen [36]. Polymer surfaces coated with human serum albumin (HSA) and fibrinogen were shown to markedly decrease neutrophil activation [35,37]. Similarly, adsorption of IGg activates complement [38,39], which, in turn, can stimulate neutrophil activation [40,41]. To the best of our knowledge, this is the first study to look at the neutrophil reaction to commercially available hydrophilic and hydrophobic titanium surfaces with the same surface topography. Within the limitations of this in vitro study, we found a significant difference in the reaction of human neutrophils to a serum-coated chemically-modified hydrophilic surface compared to a hydrophobic surface with the same surface topography. We theorize that hydrophilic surfaces are capable of faster and more favorable selective adsorption of serum proteins, which can downregulate the activation of neutrophils causing less production of oxygen radicals and tissue destruction. Further studies are needed to understand more about the type and the role that adsorbed proteins play on titanium surfaces, as well as to better understand the initial inflammatory response around implants and its impact on healing. 

## 5. Conclusions

The reaction of neutrophils to MR and CMR surfaces suggests that these surfaces, overall, provoke minimal reaction from human inflammatory cells, which could be favorable during the healing period leading to osseointegration. In the absence of exposure to serum, there were no significant differences found between hydrophilic and hydrophobic surfaces. However, after exposing both surfaces to human serum, we found was a statistically significant reduction in respiratory-burst activity by neutrophils exposed to the hydrophilic surface, suggesting a more favorable environment during the initial healing period. The results suggested that hydrophilic biomaterial surfaces might promote a more favorable initial inflammatory host response and that this response might help promote faster integration of endosseous implanted biomaterials.

## Figures and Tables

**Table 1:** Mean chemiluminescence (CL) values.

CL Measurement	Reagents (*n* = 24)	Cells Alone (*n* = 24)	Cells + MR (*n* = 24)	Cells + CMR (*n* = 24)	Cells + MR + Serum (*n* = 24)	Cells + CMR + Serum (*n* = 24)
**Active CL**	2445.349 SD (±24)	7067.98 SD (±121)	8728.43 SD (±1002)	7625.99 SD (±932)	7322.86 SD (±721)	5926.23 SD (±620)
**Mean CL**	0.45 SD (±0.038)	1.36 SD (±0.125)	1.263 SD (±0.131)	1.212 SD (±0.118)	2.392 SD (±0.25)	0.978 SD (±0.87)
**Peak CL**	0.47 SD (±0.13)	2.837 SD (±0.92)	2.791 SD (±0.89)	3.331 SD (±0.9)	3.142 SD (±0.81)	2.969 SD (±0.68)
